# Advances in cell therapies using stem cells/progenitors as a novel approach for neurovascular repair of the diabetic retina

**DOI:** 10.1186/s13287-022-03073-x

**Published:** 2022-07-30

**Authors:** Judith Lechner, Reinhold J. Medina, Noemi Lois, Alan W. Stitt

**Affiliations:** Wellcome‐Wolfson Institute for Experimental Medicine, School of Medicine, Dentistry, and Biomedical Science, Queen’s University Belfast, Belfast, UK

**Keywords:** Diabetic retinopathy, DME, PDR, Macular ischemia, Microvascular disease, Neurodegeneration, Adult stem cells, Endothelial progenitor cells, Induced pluripotent stem cells, Retinal progenitor cells

## Abstract

**Background:**

Diabetic retinopathy, a major complication of diabetes mellitus, is a leading cause of sigh-loss in working age adults. Progressive loss of integrity of the retinal neurovascular unit is a central element in the disease pathogenesis. Retinal ischemia and inflammatory processes drive interrelated pathologies such as blood retinal barrier disruption, fluid accumulation, gliosis, neuronal loss and/or aberrant neovascularisation. Current treatment options are somewhat limited to late-stages of the disease where there is already significant damage to the retinal architecture arising from degenerative, edematous and proliferative pathology. New preventive and interventional treatments to target early vasodegenerative and neurodegenerative stages of the disease are needed to ensure avoidance of sight-loss.

**Main body:**

Historically, diabetic retinopathy has been considered a primarily microvascular disease of the retina and clinically it is classified based on the presence and severity of vascular lesions. It is now known that neurodegeneration plays a significant role during the pathogenesis. Loss of neurons has been documented at early stages in pre-clinical models as well as in individuals with diabetes and, in some, even prior to the onset of clinically overt diabetic retinopathy. Recent studies suggest that some patients have a primarily neurodegenerative phenotype. Retinal pigment epithelial cells and the choroid are also affected during the disease pathogenesis and these tissues may also need to be addressed by new regenerative treatments.

Most stem cell research for diabetic retinopathy to date has focused on addressing vasculopathy. Pre-clinical and clinical studies aiming to restore damaged vasculature using vasoactive progenitors including mesenchymal stromal/stem cells, adipose stem cells, CD34^+^ cells, endothelial colony forming cells and induced pluripotent stem cell derived endothelial cells are discussed in this review. Stem cells that could replace dying neurons such as retinal progenitor cells, pluripotent stem cell derived photoreceptors and ganglion cells as well as Müller stem cells are also discussed. Finally, challenges of stem cell therapies relevant to diabetic retinopathy are considered.

**Conclusion:**

Stem cell therapies hold great potential to replace dying cells during early and even late stages of diabetic retinopathy. However, due to the presence of different phenotypes, selecting the most suitable stem cell product for individual patients will be crucial for successful treatment.

## Background

Diabetic retinopathy (DR) is the most common microvascular complication of diabetes mellitus (DM) and remains a leading cause of sight-loss in working age adults worldwide. [1, 2]. Among individuals with diabetes, the global prevalence was estimated to be 22.27% for DR and 6.17% for vision threatening DR in 2020 [3]. Diabetes is a growing global disease, and the International Diabetes Federation estimated the global population with DM to be 537 million in 2021 with a predicted estimate of 643 million by 2030 and 783 million by 2045 [4]. Hence DR will become an even more challenging health problem in the coming years and new therapeutic approaches are needed at all stages of disease.

It is well-established that diabetes causes damage to the vasculature, neurons and glia of the retina although the current clinical classification is largely based on microvascular lesions. There is now broad acceptance that retinal neurodegeneration also plays a significant role in DR [5]. This is manifest in robust neurodegenerative hallmarks such as reactive gliosis, diminished neuronal function, glutamate excitotoxicity, reduced levels of neurotrophic factors and neural accelerated apoptosis mainly affecting retinal ganglion cells and amacrine cells, although photoreceptors can also be affected [6–8]. In fact, all major cell types of the retina have been shown to be altered and DR may result from interplay between endothelial cells, neurons, microglia, astrocytes and Müller cells [9]. Furthermore, the vasculature of the choroid which supplies the outer retina, photoreceptors, and retinal pigment epithelium (RPE) undergoes changes during the course of DR which are likely to contribute to the pathology [10]. In addition, there is evidence that peripheral immune cells including neutrophils and monocytes are activated in DR leading to increased leukocyte-endothelial interaction which can cause capillary occlusion and release of pro-inflammatory cytokines, contributing to the increased vascular permeability [11, 12]. Dysfunction of the neurovascular unit is a key feature of DR but its impact on retinal function remains largely asymptomatic for patients in the early stages of their disease. As the disease develops, capillaries become progressively non-perfused resulting in retinal ischemia and hypoxia which in turn drives upregulation of pro-angiogenic factors such as vascular endothelial growth factor (VEGF) and activation of inflammatory pathways. Retinal ischemia and elevated levels of VEGF eventually lead in some patients to blood retinal barrier disruption, excessive vasopermeability, fluid accumulation and neovascularisation and ultimately to the advanced, sight-threatening stages of diabetic macular edema (DME) and proliferative DR (PDR) [2, 6, 9]. A better understanding of the underlying disease mechanisms and the interplay between intrinsic vascular cells, circulating cells and neuronal factors in retina and choroid might lead to better and more precise therapeutic approaches.

Most current treatment options such laser photocoagulation, corticosteroids, vitreoretinal surgery and anti-VEGF injections are targeted at end-stages of the disease after significant damage has already occurred [13]. Recent studies also suggest that early initiation of anti-VEGF injections, before complications of DR have occurred, can reduce the progression to severe stages [14, 15]. Anti-VEGF therapy has revolutionised the management of DR and many patients now benefit from this treatment [2]. However, not all patients respond to anti-VEGF therapy and complications can occur in some patients as a result of it [2, 16, 17]. The treatment requires being repeated regularly and long term, is inconvenient to patients, stretches the capacity of all health care systems and is expensive. [17]. At present there are very limited therapeutic options available to prevent progression from the early stages of DR to sight-threatening DR. Fenofibrate seems to be promising for this purpose [18] and it is licenced in some countries to prevent progression of DR. However, further evidence is needed [19]. Other options to reduce the risk of developing DR or arresting its progression include tight control of glycemia, dyslipidemia and hypertension [2]. Neuroprotective therapies also hold potential for the management of DR and several pre-clinical studies in diabetic mouse and rat models have shown beneficial effects such as reduced neuronal cell death and glutamate excitotoxicity when administering neuroprotective agents including insulin-like growth factor 1 (IGF-1), pigment epithelium-derived factor (PEDF), somatostatin, pituitary adenylate-cyclase-activating polypeptide (PACAP), glucagon-like peptide-1 (GLP-1) and neurotrophins such as brain-derived neurotrophic factor (BDNF) and nerve growth factor (NGF) [20]. Although a clinical trial using two potential neuroprotective strategies, brimonidine and somatostatin, did not detect differences between groups and when compared with placebo, these drugs appeared to prevent progression of retinal functional changes in people with pre-existing neurodysfunction [21].

There is a need for efficacious new therapies at both early and late stages of DR. Once cell loss has occurred in DR, stem cell therapies may be able to restore them, and if risks associated with their use were to be low, these could be administered even in early stages of disease to people at higher risk of developing sight-threatening complications.

The potential of regenerative therapies for the treatment of DR are currently explored by many investigators [22, 23]. The hypothesis that retinal function can be restored through repairing retinal blood flow is supported by case reports of spontaneous reperfusion of an ischemic retina and recovery of visual acuity [24]. Spontaneous reperfusion has also been reported in the ischemic diabetic retina [25, 26]. Muraoka et al. reported a relatively high incidence of revascularisation of nonperfused areas (in 40 of 60 eyes with DR) through repeated fluorescein angiography [27]. Vessel resident endothelial progenitor cells are important for maintaining vascular homeostasis and promoting vascular repair in pathological situations. Upon vascular injury, progenitors can become activated and contribute to endothelial regeneration and promote restoration of perfusion [28–30]. Lineage tracing experiments in mice revealed that vessel-resident endothelial progenitors participate in neovessel formation during wound healing [31]. Chronic wounds due to impaired wound healing are well documented in diabetes mellitus [32] indicating deficiencies in normal repair processes suggesting that endogenous vascular repair mechanisms might also be compromised. Enhancing or restoring endogenous repair mechanisms might present a novel therapeutic angle [33]. In contrast, replacing dying cells with stem cell therapy holds potential for restorative therapeutic approaches in DR [22, 23]. Several different stem cells have been tested in pre-clinical models of DR including embryonic or induced pluripotent stem cells (iPSC), hematopoietic stem cells, endothelial progenitor cells (EPCs), and mesenchymal stromal cells (MSC) [22]. However, due to the complex nature of DR, selection of the most promising stem cell product remains a challenge. Furthermore, determining the patients most at risk of sight-loss and those that would benefit most from restorative therapy is still difficult. Finally, more research is needed to determine the exact time point to initiate treatment to obtain best outcomes.

## Clinical classification of diabetic retinopathy

**Fig. 1:**
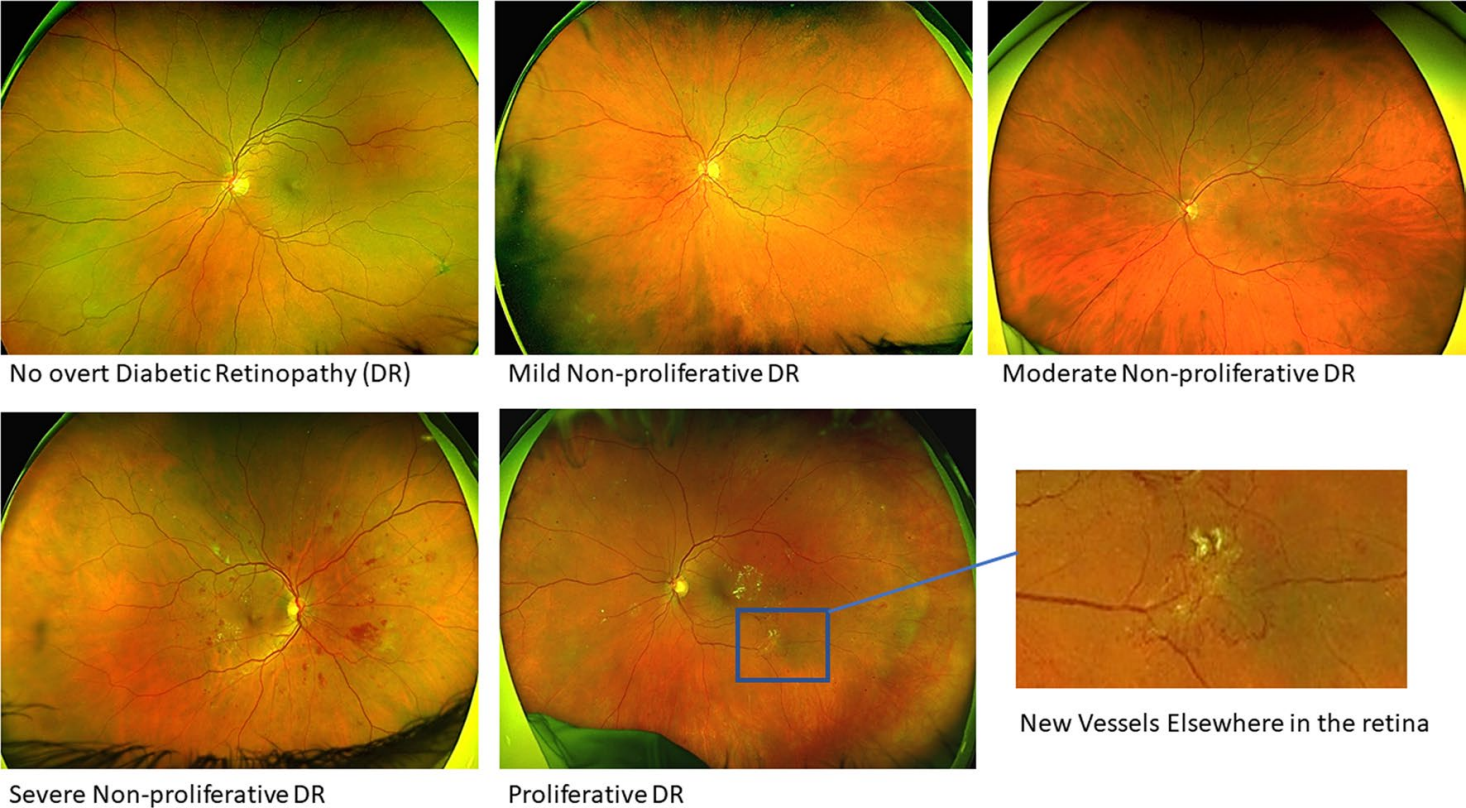
Ultra-widefield fundus images showing five stages of Diabetic Retinopathy (DR) according to the international classification of DR

Clinically, DR is classified based on anatomical features and the presence and severity of vascular lesions as detectable on fundus photographs (see Fig. 1).

The first level of DR is “no retinopathy” (referring to no “overt” retinopathy as seen in retinal fundus images).The second level “mild NPDR”, which is characterised by microaneurysms only. The risk of significant progression over several years is very low in both groups. The third level, “moderate NPDR,” is characterised by more than just microaneurysms, e.g. presence of intraretinal hemorrhages and/or hard exudates, and/or venous beading but less than severe NPDR;the risk of progression in this group increases significantly. The fourth level, “severe NPDR” is characterised by any of the following: more than 20 intraretinal hemorrhages in each of four quadrants; venous beading in at least two quadrants andintraretinal microvascular abnormalities (IRMA) in at least one quadrant with no signs of proliferative retinopathy (PDR). The hallmark of the fifth level, “PDR”, is the presence of new vessels in the optic disc or elsewhere in the retina.

Diabetic macular edema (DME) can occur at any stage of DR DME can beclassified as “mild”, “moderate” or “severe depending on the distance from the centre of the fovea of retinal thickening and/or hard exudates [34]. DME is evaluated clinically on slit-lamp biomicroscopy and optical coherence tomography (OCT).

## Retinal changes prior to the clinical onset of diabetic retinopathy

There is an early stage in DR when there are functional and structural changes in the retina that are not detected on routine fundus examination or fundus photography. Fundus fluorescein angiography (FFA) performed in eyes of individuals with diabetes but without clinically visible (i.e. “overt”) retinopathy may reveal early retinal vascular changes present in more than half of the eyes studied including dye leakage, dilatation of capillaries and changes in retinal blood flow [35, 36]. When using ultra-wide field imaging with and Optos imaging system (Dunfermline, Scotland, UK) only 1.5 millilitres of fluorescein dye are needed to obtain high quality fluorescein angiograms depicting the entire retinal vascular tree. These findings have been noted also using optical coherence tomography angiography (OCT-A) [37, 38]. OCT-A is a non-invasive imaging technique able to capture the microvasculature of the retina without the need of an injectable dye.With OCT-A it is possible to visualise superficial and deep retinal vascular plexuses which was previously only achievable on histological examinations [39]. OCT-A allows a better understanding of how capillaries change over the course of DM and has been used successfully in several studies to detect early microvascular changes in individuals with diabetes but without overt retinopathy [37, 38]. With the availability of ultra-widefield imaging, peripheral retinal pathology, missed by traditional angiography, can be detected [40]. Studies have also shown that the presence and extent of peripheral lesions in individuals with DR are associated with an increased risk of DR progression and severity [41].

Spectral-domain optical coherence tomography (SD-OCT) provides anatomical and structural information and has been used to investigate neurodegenerative changes such as ganglion cell complex (GCC) thickness, a measure of ganglion cell loss. While some studies identified reduced GCC thickness in individuals with diabetes prior to the onset of overt DR compared to healthy controls [42, 43], other studies could not find any significant differences [44, 45]. Disorganisation of retinal inner layers (DRIL) detectable by SD-OCT has been shown to be a more reliable marker to predict visual acuity and DR severity [46, 47]. DRIL has been detected in some individuals with diabetes without overt DR [48].

In addition to structural changes of the neuroretina, functional changes prior to the onset of clinically overt DR have been reported as alterations in oscillatory potentials in the electroretinogram (ERG), a measure of outer retinal function [49, 50]. Neuronal dysfunction has further been demonstrated by studies using multifocal ERG (mfERG) in individuals with diabetes without overt DR as early as two years after diagnosis of DM [51, 52]. Even in individuals with prediabetes (glycated haemoglobin (HbA1C) level between 5.7 and 6.4%) ERG responses were reduced compared to normal controls (HbA1C level below 5.7%) [53]. Functional assessments including visual acuity, contrast sensitivity and perimetry have also shown functional impairments in individuals with diabetes prior to the onset of clinically over DR when compared to individuals without DM [54].

**Fig. 2:**
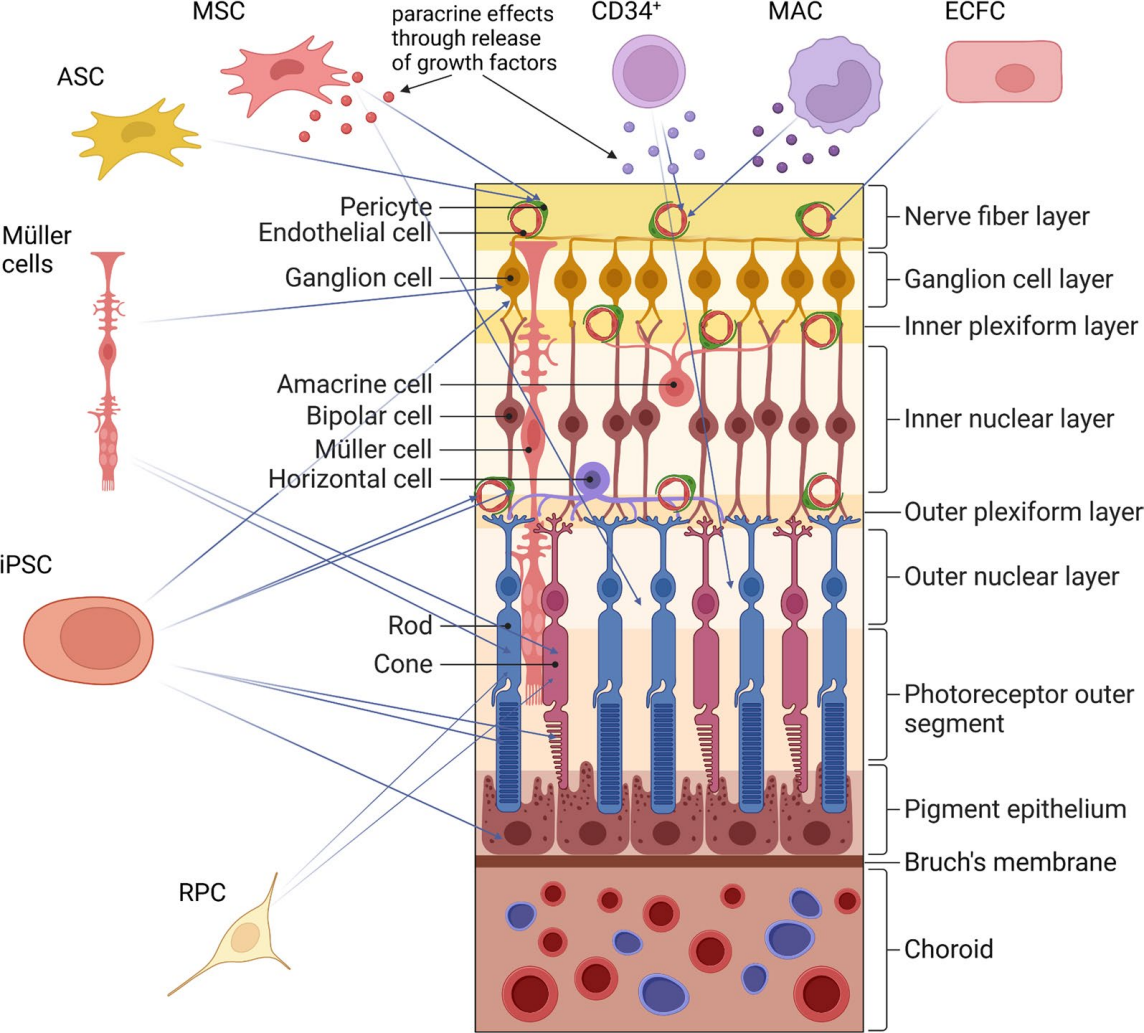
Structure of the retina and overview of various stem cells that may be used to restore damaged cells in Diabetic Retinopathy. *ECFC* Endothelial colony forming cells; *MAC* Myeloid angiogenic cells; *MSC* Mesenchymal stromal/stem cells; *ASC* Adipose stromal/stem cells; *iPSC* Induced pluripotent stem cells; *RPC* Retinal progenitor cells. Schematic created with BioRender.com

The European Consortium for the Ealy Treatment of Diabetic Retinopathy (EUROCONDOR) study found that 61% of patients with diabetes without visible microvascular disease presented neurodegenerative abnormalities detected by mfERG and SD-OCT. When assessing patients with visible but mild microvascular disease, 68% of patients showed neurodenerative changes while 32% did not present any functional or structural neurodegenerative abnormalities. These findings suggest that some patients might have a primarily neurodegenerative phenotype while others a primarily microvascular phenotype [7]. However, FFA was not performed in this study and might have identified early vascular changes in at least a proportion of the 61% of patients labelled as having”no DR”. Similarly, ultra-widefield OCT and full field ERG might have identified retinal neurodegeneration in at least some of the 32% of patients reported with mild microvascular disease only. Further studies, thus, are needed to confirm the existence of two distinct phenotypes e.g. primarily microvascular vs primarily neurodegenerative phenotype at the beginning of DR pathology. A better understanding of different disease phenotypes could have implications for therapy as some patients might benefit more from neuroprotective rather than vasoprotective agents at early stages of the disease. While most attention has been focused on stem cells addressing vasculopathy for the treatment of DR, restoring other damaged cells including neurons, RPE and glia cells might have beneficial effects especially in patients that do not have a primarily microvascular phenotype (see Fig. 2).

## Stem cells for retinal vasculopathy

The microvasculature of the retina is made up of endothelial cells and pericytes and provides the required nutrients to the neuronal tissue. Tight junctions between endothelial cells maintain the inner blood retinal barrier (iBRB) which is tightly controlled by signaling of all components of the retinal neurovascular unit including neural and glial cells along with pericytes and retina resident immune cells [2, 9]. Altered blood vessel permeability and breakdown of the iBRB due to abnormal pericyte-endothelial interactions, loss of pericytes and activated circulating immune cells are among the first microvascular abnormalities that occur because of diabetes. Pericytes wrap around retinal capillaries to provide structural and functional support. Their importance in the retinal microvasculature is highlighted by the high ratio of retinal pericytes to endothelial cells (1:1) [55]. Loss of pericytes promotes increased VEGF signaling and permeability and eventually leads to loss of endothelial cells [2, 6].

While embryonic stem cells can create every cell in the body (pluripotent) and are capable of unlimited self-replication, adult stem cells have significant albeit limited self-renewal potential, are committed to generate cell types of a specific lineage (multipotent) and are responsible for tissue maintenance and repair of the tissue they reside in e.g. endothelium. Progenitor cells are descendants of stem cells that are committed to targeted differentiation into specialized cell types and it has been suggested that endothelial progenitor cell niches exist within highly vascularized organs [56]. The identification of these rare progenitor cells in many adult tissues opened new therapeutic avenues for degenerative diseases [57]. Stem/progenitor cell therapy may be an attractive therapeutic option to replace lost endothelial cells and pericytes in DR to restore perfusion and vascular function.

Mesenchymal stromal cells (MSC) are among the most intensely studied cell type in cell therapy and can be isolated from various tissue sources including bone marrow, adipose tissue, dental origin, placenta, peripheral blood and umbilical cord blood [58]. They are defined as multipotent progenitor cells with fibroblast-like spindle shape morphology, trilineage mesenchymal differentiation capacity (adipocytes, osteoblasts and chondroblasts), strong adherence to plastic, expression of specific cell surface markers and high proliferation capacity [59] and therefore hold great potential for regenerative medicine. Moreover, in vitro studies have demonstrated MSCs can acquire endothelial like markers [60, 61]. MSCs secrete a broad range of growth factors and their main mode of action is through paracrine and trophic mechanisms to promote cell repair and protect against cell stress damage [62]. MSCs have been used as therapeutic agents in several preclinical models with a broad range of beneficial effects including vascular protective effects [63]. In the oxygen-induced retinopathy (OIR) mouse model, intravitreally injected bone marrow derived MSCs (BMSCs) showed beneficial vascular effects by reducing the avascular area and neovascularization [64, 65]. MSC increased blood perfusion in the hind limb ischemia model and their angiogenic therapeutic potential was further enhanced when MSCs were induced to express endothelial markers in vitro prior to cell transplantation [66]. In a recent study, MSC where isolated from umbilical cord Wharton’s jelly and induced to differentiate into endothelial like-cells. Six weeks after STZ diabetes induction in old rats, endothelial like MSCs were injected intravenously and were able to restore altered vascular functions [67].

Several vasoactive progenitors, including CD34^+^ cells, have been shown to enhance repair of ischemic tissues in the retina of preclinical models including streptozotocin (STZ) induced diabetic mouse model [68, 69], retinal ischemia–reperfusion (I/R) injury mouse model [70, 71] and OIR mouse model [71]. Bone marrow contains the highest concentration of CD34^+^ cells however, they have also been isolated from umbilical cord blood, adipose tissue and fetal liver [72]. While CD34 is the most frequently used marker to identify endothelial progenitors, it is also expressed by hematopoietic stem cells, differentiated endothelial cells and mesenchymal stem cells [73]. As a result, isolated CD34^+^ cells represent a poorly defined heterogeneous mixture of cells. Nonetheless, studies using CD34^+^ cells have shown beneficial effects, most likely through paracrine mechanisms, in preclinical models of DR. Human CD34^+^ cells isolated from peripheral blood [71] or bone marrow [68, 69] were injected intravitreally into STZ-induced diabetic mice and were able to home to the damaged tissue and preserve the retinal vasculature. Microarray analysis of the retina one week after CD34^+^ cell injection revealed gene expression changes in pathways related to the pathogenesis of DR including inflammatory pathways such as Toll-like receptor, MAP kinase, oxidative stress, cellular development, assembly and organization pathways [68]. Intravitreally injected GMP-grade human CD34^+^ cells in immunodeficient mice were detectable in the retinal vasculature four months after injection and the study reported no major safety concerns [70]. A currently ongoing phase I clinical trial (NCT01736059) using intravitreally injected autologous bone marrow-derived CD34^+^ cells in eyes with ischemic or degenerative retinal conditions has not found any local or systemic adverse effects or major safety concerns. A larger prospective study with longer follow-up is planned to further explore the safety and potential efficacy of this cellular therapy [74].

Myeloid angiogenic cells (MACs) also known as early endothelial progenitor cells (early EPCs) or circulating angiogenic cells (CACs) do not have significant proliferative capacity but have pro-angiogenic effects through paracrine mechanisms [75]. MACs injected intravitreally in the OIR mouse model contributed to vascular repair of the ischemic retina by inducing intraretinal angiogenesis and reperfusion without incorporating into the endothelium [76].

Endothelial colony forming cells (ECFCs), also known as outgrowth endothelial cells (OECs) or late EPCs, are a type of endothelial progenitor cell that are isolated from peripheral adult blood or umbilical cord blood. They lack hematopoietic markers (CD45 and CD14) but possess endothelial markers (CD31, CD146, VEGFR2) and are committed to endothelial lineage [75, 77]. In vitro ECFCs are characterised by a high proliferative and tube-forming potential [78]. Most importantly they can incorporate into pre-existing capillaries in vivo as demonstrated in the OIR mouse model [78, 79]. In the diabetic Ins2Akita mouse model, intravitreally injected ECFCs, combined with recombinant angiopoietin 1 gene therapy, integrated into the ischemic vasculature and prevented vision loss [80]. ECFCs can induce vasoprotective effects not only through directly incorporating into the damaged vasculature but also through paracrine mechanisms such as the release of pro-angiogenic factors [81] or the secretion of extracellular vesicles [82, 83]. Furthermore, they have been shown to provide support to other reparative cells. Administration of ECFCs together with mesenchymal progenitor cells (MPC) in the hind limb ischemia model restored blood flow to a greater extent than ECFCs or MPCs administered alone [84]. In human, combined administration of ECFCs and MSC accelerated the healing of diabetic refractory wounds [85].

Induced pluripotent stem cells (iPSCs) are derived from mature cells such as dermal fibroblasts reverted to pluripotency by retrovirus techniques and expression of reprogramming factors [86]. iPSCs can be reprogrammed to differentiate into any of the three germ layers and as a result hold great promise in the field of regenerative medicine. However, the potential of iPSCs to generate teratomas represents a medical risk [87] and therefore iPSCs that have reached a certain differentiation stage, such as iPSC derived vascular or vascular progenitor cells may provide a safer option [88]. Human iPSC derived endothelial cells (hiPSC-ECs) showed greater angiogenic potential when compared to mature human retinal endothelial cells (HRECs) in response to hypoxia in vitro. Furthermore, in the OIR mouse model, hiPSC-EC injected intravitreally incorporated into the regenerating retinal vessels and promoted vascular recovery as well as reduction in ischemic retinal area [89]. Prasain et al. optimised a protocol for differentiation of hiPSCs into ECFCs. These hiPSC-ECFCs displayed characteristic cobblestone morphology and 3D tube forming potential in Matrigel similar to cord blood derived ECFCs (CB-ECFCs). Furthermore, hiPSC-ECFCs demonstrated in vivo vessel-forming capacity and did not form teratomas after more than 6 months of implantation into immunodeficient mice. In both the OIR mouse model and hind limb ischemia model hiPSC-ECFCs contributed to vascular repair similar to CB-ECFCs [79]. Vascular progenitor cells (CD31^+^ CD146^+^) generated from cord blood derived hiPSCs (CB-iPSC-VP) migrated into deep retinal layers and incorporated into damaged retinal vessel in the retinal I/R injury mouse model. Engrafted CB-iPSC-VP could be detected long term for at least 45 days [90]. Interestingly, CB-iPSC-VP were detected engrafting in both luminal endothelial and abluminal pericytic locations. The location of engraftment was influenced by the route of administration: intravitreal injection primarily resulted in homing to pericytic locations while intravenous injection largely led to homing at endothelial positions [90]. These findings suggest that CB-iPSC-VP may give rise not only to endothelial cells but pericytes as well, an exciting therapeutic prospect given the important role of pericytes to maintain BRB in the retinal microvasculature.

While most effort has been focused on replacing endothelial cells to promote vascular repair, protocols have been optimised to generate cells expressing characteristic pericyte markers, including CD146, Neuron-glial antigen 2 (NG2), and Platelet-derived growth factor receptor *beta* (PDGFR-β) from hiPSC [91] and their therapeutic potential has been demonstrated in in vitro and in vivo models. Transplantation of iPSC derived pericytes in the murine ischemic limb model promoted vascular repair and muscle recovery [92]. In vitro co-culture models demonstrated iPSC derived pericytes were able to restore transendothelial electrical resistance (TEER) in stressed brain microvascular endothelial cells through the secretion of soluble factor [93].

Adipose stem cells (ASC), a type of mesenchymal stromal/stem cell harvested from adipose tissue have been shown to have pericyte characteristics and express pericyte markers including α-smooth muscle actin (α-SMA), PDGFR and NG2 [94–96]. When injected intravitreally in the OIR mouse model, human ASCs promoted vessel regrowth and were able to integrate with the retinal microvasculature and co-localise with capillaries at pericyte-specific positions [96, 97]. Some cells remained engrafted eight weeks post injection and retained expression of pericyte markers. Injection of human ASCs prior to OIR led to a decrease in avascular area indicating their role in stabilizing the retinal microvasculature. The same group also showed that isolated murine ASC-derived pericytes (α-SMA^+^ PDGFR^+^) from epididymal fat pad integrated and associated with the retinal microvasculature when intravitreally injected in the Akimba mouse model of diabetic retinopathy. Vascular drop out was significantly reduced in retinas treated with murine ASC [96]. Similarly, in the STZ-induced diabetic rat model, human ASCs colocalised with the retinal host vasculature at perivascular positions seven days after intravitreal injection and remained there for at least 21 days. Diabetic rat retinas that received ASC-derived pericytes showed decreased BRB breakdown and retinal endothelial cell apoptosis, restored ERG responses and down-regulation of inflammatory gene expression compared to saline injected retinas [98].

## Stem cells for retinal neuropathy

The retina contains five different types of neurons including photoreceptors that transform light signals into biologic signals, second order neurons (bipolar, horizontal and amacrine cells) that relay the signal and ganglion cells that transport the visual signal through the optic nerve to the visual cortex in the brain while glia cells (Müller cells and astrocytes) provide metabolic and structural support [8, 9]. Müller cells are the main glia in the retina and span the entire width of the retina allowing them to be in contact with retinal vasculature as well as neurons [99]. It has become clear in the literature that neurodegeneration is involved in the pathogenesis of DR and that some patients might have a primarily neurodegenerative phenotype as opposed to a primarily microvascular phenotype [7, 8]. Some individuals with diabetes have thinner neural layers due to loss of neurons by apoptosis even before the onset of DR [8, 42]. The first neurons affected by diabetes-induced apoptosis are ganglion and amacrine cells, but photoreceptors are also affected [8]. Apoptosis of ganglion cells was detected in the mouse retina as early as 10 weeks after STZ-diabetes induction [100] and in the type 2 diabetes mouse model (db/db) from 20 weeks of age [101]. In the STZ-induced diabetic rat retina, apoptosis of photoreceptors was notable from four weeks after the onset of diabetes [102, 103]. Apoptosis of cone photoreceptors was detected in post mortem retinal tissues of patients with varying degrees of DR [104] as well as in three month old diabetic Ins2Akita mice [105]. Müller cells become activated in diabetes as evidenced by increased expression of glial fibrillary acidic protein (GFAP), a common marker of reactive gliosis. As a result, Müller cells release growth factors such as VEGF and cytokines that in the long term contribute to neuronal dysfunction [99]. Therapeutic approaches including neuroprotection and neuronal cell replacement therapy have the potential to be an alternative treatment for DR especially in cases with a primarily neurodegenerative phenotype [7]. Most neuronal stem cell studies to date have focused on the generation and replacement of photoreceptors [106] and retinal ganglion cells [107]. However, the generation of new functional synaptic connections between transplanted donor cells and remaining host neurons remains a major challenge in the field [108].

As mentioned in the previous section, MSC secrete a broad range of growth factors including neurotrophic factors such as nerve growth factor (NGF) and brain-derived neurotrophic factor (BDNF) [62]. Intravitreal injection of MSCs in the STZ-induced diabetic mouse model increased ocular levels of neurotrophic factors, reduced lipid peroxidation levels and prevented ganglion cell loss at four and 12 weeks after MSC administration [109]. BMSCs were able to reduce levels of gliosis and improve ERG responses in STZ-induced diabetic rats three weeks after intravitreal injection [110]. Beneficial results were also observed in clinical studies where Wharton’s jelly derived MSCs injected into the subtenon space of the eye of retinitis pigmentosa patients improved visual acuity, outer retinal thickness and mfERG results six months after implantation. Importantly, no serious adverse events or immune rejections were detected during the six months follow up [111]. Human bone marrow derived CD34^+^ cells were also shown to have trophic neuroprotective effects in addition to vasoprotective effects when injected intravitreally or subretinally into retinal dystrophic rats. Functional improvements were reported two weeks after cell transplantation including increased b-wave amplitude as assessed by ERG and preservation of the outer nuclear layer as assessed by histology. However, improvements were transient and not detectable seven weeks after transplantation [112].

Retinal progenitor cells (RPCs) are usually derived from fetal retinas, embryonic stem cells (ESCs), and induced pluripotent stem cells (iPSCs). The human retina at 12 to 18 weeks of gestation contains progenitor cells that can be isolated and expanded in vitro. The isolated cells express retinal stem cell markers nestin, Ki-67, PAX6 and LHX2 as well as photoreceptor markers (recoverin, blue opsin and rhodopsin) after differentiation [113–115]. Functional analysis of RPCs indicated typical neural phenotype as cells responded with calcium influx following neurotransmitter stimulation (glutamate) [115]. Human RPCs injected subretinally into the mouse eye were able to survive, migrate and integrate with the host retina. A small number of transplanted cells expressed rhodopsin indicating differentiation into mature photoreceptors in vivo [113]. Further studies in retinitis pigmentosa animal models confirmed that transplanted cells were able to integrate into the host retinas, differentiate into photoreceptors and rescue vision [116–118]. However, subsequent studies indicated that visual improvement may have been a consequence of cell fusion or cytoplasmic material transfer from donor to host photoreceptors to partially restore their function rather than structural integration of donor cells [119–122]. Nonetheless, synaptic connections between donor and host cells have been demonstrated in an end-stage retinal degeneration model (rd1 mouse) where the outer nuclear layer (ONL) of photoreceptors had been lost prior to donor cell transplantation [123, 124]. Efficacy and safety of human RPCs was demonstrated after transplantation of human RPCs in wild type and dystrophic rat retinas where no uncontrolled cell growth, tumor formation or unexpected alterations to retinal structure were found six months after transplantation. Visual improvements were detected by optokinetic response measurements as well as increased ONL thickness, however both were more pronounced at earlier time points after transplantation [125]. Interestingly, improved visual function was longer maintained when human RPCs were transplanted together with human bone marrow-derived mesenchymal stem cells [126]. Fetal-derived RPC transplantation in retinitis pigmentosa patients showed improvements in visual acuity between two and six months after surgery, however, no differences were detectable by 24 months. Nevertheless, the clinical study demonstrated that human RPCs were safe and tolerated in human without immunological rejection or tumor formation [127].

Due to the limited ability of human RPCs to expand in culture [113] as well as significant ethical concerns, legal and logistical challenges, protocols have been developed in recent years using a range of inhibitors and growth factors to generate photoreceptors from ESC and iPSC [128–132]. Interestingly, ESC and iPSC can self-organize and generate optic cups in a three-dimensional culture system. This technology not only increases photoreceptor yield and reproducibility but also provides a powerful platform for disease modelling and translational studies [133]. Pre-clinical studies using ESC- and iPSC-derived photoreceptors have demonstrated beneficial effects in a variety of animal models [129, 130, 134, 135]. Human ESC-derived retinal progenitors, transplanted into the subretinal space of adult mice were able to migrate into the ONL and expressed photoreceptor specific markers such as recoverin and rhodopsin. To examine the functionality of hESC-derived retinal progenitors, they were transplanted into the subretinal space of adult Crx − / − mice, a model of Leber's Congenital Amaurosis characterised by photoreceptor degeneration and defective ERG response. Two to three weeks after transplantation, ERG traces showed some response to light in the injected eye while the non-injected eye had no response to light. Interestingly, the size of the transplanted area correlated with the b-wave response and in injected eyes where no cells were found, the ERG response was absent. Even though hESC were differentiated to retinal cells prior to transplantation, there is a risk of teratomas formation arising from undifferentiated cells. No teratomas were found in any of the mice that received transplanted cells suggesting no undifferentiated cells had remained after the retina directed cell culture differentiation [130]. In the dystrophic rat model, injected hESC-derived photoreceptors survived for up to eight weeks and optokinetic response was preserved for up to 20 weeks [129]. Another study using the rd1 mouse model demonstrated that despite a relatively low engraftment yield of hESC-derived photoreceptor precursors (1.5% of cells integrated into the host ONL) visual improvements were observed in two different visual behavioural tests. Furthermore, transplanted photoreceptors were able to elicit light responses as measured by *ex-vivo* multielectrode array retinal recordings whereas light responses were absent in sham injected retinas [136]. Photoreceptor progenitors derived from hiPSC survived up to three weeks post transplantation into the subretinal space of the rd1 mouse model, differentiated to mature photoreceptors and connected to host retinal neurons. Two different visual behavioural tests showed visual improvements in treated animals [124]. Promising results were obtained from studies in non-human primates where photoreceptor precursors derived from hESC [134] or hiPSC [135] were injected subretinally following photoreceptor ablation. Donor cells were able to survive and differentiate into mature photoreceptors, form synaptic connections with host bipolar cells and mediate structural recovery.

There is evidence that Müller cells have neural stem cell properties and therefore present an attractive source for use in cell therapies [137–139]. In zebrafish, Müller cells undergo reprogramming upon injury and proliferate to progenitor cells leading to retinal repair and restoration of vision [140]. Müller stem cells were also described in the human adult retina but their regeneration potential is limited [141]. However, Müller cells isolated from mouse and human retinas were able to differentiate into neuronal progenitors and photoreceptors during in vitro culture conditions [142–144]. Transplantation of Müller glia-derived photoreceptors into neonatal mouse eyes revealed they can survive, integrate in the retina and express rod-specific markers [143]. Furthermore, functional improvements were demonstrated when Müller cell derived photoreceptor precursors were transplanted into the retinas of P23H rats, a model of rapid photoreceptor degeneration. Transplanted donor cells migrated and integrated within the host outer nuclear layer. ERG performed four weeks after transplantation revealed improved responses in eyes transplanted with differentiated Müller cells compared to eyes transplanted with undifferentiated cells [145].

In addition to photoreceptor precursors, Müller cells have also been successfully differentiated into ganglion precursors under defined in vitro culture conditions. Human Müller cell derived ganglion cell precursors, transplanted intravitreally into rats that were previously depleted of ganglion cells, integrated into the host retina, localized to the retinal ganglion cell (RGC) layer and expressed RGC markers four weeks post transplantation. Partial functional restoration was observed in transplanted eyes as assessed by ganglion specific ERG responses [146]. Similar functional ganglion cell improvements were observed after transplantation of Müller glia derived from retinal organoids formed by hiPSC into the same rat model of ganglion cell depletion [147]. Other cellular sources for the generation of ganglion cells include human ESC and iPSC [148–151]. Ganglion cells derived from human ESC under carefully defined culture conditions formed extended long neurites, demonstrated a similar transcriptome profile as human ganglion cells in vivo and demonstrated functional electrophysiological profiles as well as axonal transport of mitochondria [148]. Human ESC derived neuronal progenitors injected intravitreally into ganglion cell depleted mice were able to survive and differentiate to RGC lineage. Visual acuity was improved in mice two months after transplantation as demonstrated by optokinetic response measurements [149]. While not investigated in the context of DR, mesenchymal stem cells could be a potential therapy for retinal ganglion cells due to their release of neuroprotective factors as demonstrated in animal models of glaucoma [152, 153].

## Stem cells for replacement of the retinal pigment epithelium

There is evidence that changes to the retinal pigment epithelium (RPE), located between the choriocapillaris and neurosensory retina, contribute to the development of vascular lesions in DR [6]. Tight junctions between the epithelial cells form the outer blood-retinal barrier (oBRB) that controls the movement of fluid and metabolites into and out of the neural retina [6, 9]. These tight junctions are compromised in diabetes and breakdown of the RPE leads to leakage into the neuroretina from the choroid as demonstrated in diabetic mice and rats by fluorescent microscopy after intravenous injection with fluorescein isothiocyanate (FITC)-dextran [154]. Fluorescein leakage diffused from the RPE has also been demonstrated in patients with early stages of NPDR [64]. The RPE actively removes water from the subretinal space and plays a critical role in the fluid homeostasis of the outer retina and choroid [155]. Increased fluid entry into the retina or decreased drainage function can contribute to the development of DME [156]. The RPE is also crucial for maintaining the structural and functional integrity of the photoreceptors by supplying essential nutrients and removing metabolic waste products [157]. This transcellular transport is compromised in diabetes contributing to DR pathology in the neuroretina even in early stages of DR [157]. Most RPE stem cell research to date has been conducted in the context of Age-related Macular Degeneration (AMD) as RPE dysfunction is the main characteristic in this pathology [158] and further investigation is needed to assess whether RPE replacement therapy might have beneficial effects in DR. RPE cells are easily maintained and unlike photoreceptors do not form synaptic connections and therefore present a promising target for replacement therapy.

RPE cells have successfully been derived from human embryonic stem cells (hESCs) as well as from hiPSC [159–161]. Human ESCs-derived RPEs demonstrated RPE specific marker expression as well as active phagocytosis of photoreceptor outer segments. Importantly, no tumor formation or migration to organs was detected for up to seven months after subcutaneous injection into immunodeficient mice. Furthermore hESC‐RPE were detectable in the rabbit subretinal space at four weeks after subretinal injection [162]. Additional preclinical studies in animal models of retinal degeneration demonstrated that hESC-RPEs were able to protect photoreceptors and rescue retinal function [161, 163]. Transplantation of a hESC-derived RPE patch in two dry AMD patients improved vision for at least 12 months as observed in a phase 1 clinical trial. The study further demonstrated feasibility and safety of hESC-RPE patch transplantation. However local long-term immunosuppression was required [164]. Subretinal transplantation of hESC-RPE cells has also been performed in a clinical trial in patients with Stargardt disease with promising results and no adverse effects due to the stem cell therapy [165].

Human iPSC derived RPE cells have been generated successfully and were comparable to native RPE cells in terms of polarisation, phagocytic activity and gene expression pattern [159]. Their phenotype was further characterised and additional RPE specific functions such as polarised VEGF secretion, ion channel activity and membrane potential were demonstrated [160]. However, hiPSC-RPE displayed fast telomere shortening, DNA chromosomal damage, increased p21 expression and growth arrest [160] which may negatively affect the survival of transplanted cells especially when placed in a hostile microenvironment as is the case in the DR retina. Nonetheless, preclinical studies have demonstrated beneficial effects of transplanted hiPSC-RPE. Subretinal space transplantation of hiPSC-RPE cells into the retina of retinal degeneration 10 (rd10) mice [166] or retinal dystrophic rats [167] had beneficial effects including preserving retinal structure, reducing inflammation, removing retinal debris and improving visual function. A long-term preclinical safety study reported no tumor formation or abnormal proliferation after subretinal injection of iPSC-RPE cells in immunodeficient mice until 15 weeks post surgery. In addition, transplanted hiPSC-RPE cells survived and rescued the visual function for at least 15 weeks post surgery under immunosuppressive conditions in the retinal dystrophic rat model [168]. In a clinical study, where an iPSC-RPE graft sheet, derived from the patient’s skin fibroblasts, was transplanted for the treatment of AMD, no serious complications were reported and no unexpected proliferation or sign of local or systemic malignant disease was observed. The patient’s visual acuity remained the same after the transplantation, however the self-reported vision-targeted health status improved [169].

## Challenges of stem cell therapy for diabetic retinopathy

Despite significant progress made in recent years in the field of stem cell therapies to treat diabetic eye disease, several challenges remain that need to be addressed. One major hurdle for any stem cell therapy for DR is the hostile microenvironment present in the retina [2, 9] that besides negatively affecting endogenous repair mechanisms, can also impact homing and survival of transplanted donor cells. Genetically engineered stem cells that express factors to make cells more resilient may be useful to overcome these challenges [170, 171]. Additionally, the impact of the hostile microenvironment may be minimised by treating at earlier stages of the disease. Initiating treatment early, however, would require the identification of individuals at higher risk of sight-loss to justify “prophylactic” therapy. Due to technical advancements, currently available clinical diagnostic technologies including OCT, OCT-A (which allows evaluation of superficial and deep retinal capillary plexuses) [39], ultra-widefield imaging [40], rapid electrophysiology testing (e.g. with systems such as the RETeval) [172], macular microperimentry, and fundus-mapped visual field testing [173] allow more appropriate phenotyping of patients. However, imaging of the choroid and choriocapillaris remains challenging [174]. While progress has been made to better understand the underlying pathogenesis of DR, this remains unclear [6]. Recent reports of potentially distinct phenotypes among patients with DR (e.g. primarily microvascular degenerative phenotype vs primarily neurodegenerative phenotype), highlights the importance of choosing the right stem cell therapy product for individual patients in order to regenerate the predominantly affected cells [7]. Due to the multifactorial nature of DR, combining different stem cells might have beneficial effects for the management of DR [126]. Furthermore, systemic disease factors such as circulating immune cells, blood glucose and lipid levels and blood pressure might have an impact on the success of any treatment. An attractive alternative to stem cell therapy is to protect and reactivate endogenous stem/progenitor cells and endogenous repair mechanisms to enable the mammalian retina to repair itself. Such therapies may be less time consuming and may present fewer complications such as immune rejection. Several pathways have been identified that might be targeted to activate endogenous repair mechanisms, but further research is needed to confirm their potential clinical application [175]. Since DR is a disease that develops over decades, finding the best time point for stem cell transplantation or initiation of endogenous repair to promote retinal regeneration is critical. On the other hand, the slow process of disease progression presents a longer time window for treatment before sight-threatening end-stages are reached. Finally, any new treatment should be easy to deliver, not expensive and with low risk of complications. In addition to assessing tumorigenesis of stem cell therapies, host immune responses and cell biodistribution, as well as other potential complications related to its delivery, need to be addressed to ensure the cell therapy product is safe and without serious adverse side effects. Proof of efficacy and safety as well as appropriate regulatory oversight of any new treatment are essential to avoid detrimental outcomes for patients [176]. Further research is needed to address these challenges and to translate restorative stem cell therapies into the clinic.

## Conclusions

During early stages of DR there is progressive degeneration of the retinal neurovascular unit due to prolonged hyperglycemia. Current treatment options target mainly end-stages of the disease (i.e. DME and PDR) and there is an opportunity to address the progressive loss of vascular and/or neuronal cells earlier in the disease process by enhancing endogenous repair mechanisms or replacing dying cells through stem cell therapies. Even patients with complications of DR (macular ischemia, DME and PDR) might benefit from stem cell therapies to restore damaged/lost cells. It is essential to be able to identify individuals with diabetes that would benefit most from such therapies and determine when they should be offered. Future research is needed to advance our knowledge of underlying disease mechanisms and to translate findings clinically.

## Data Availability

All data generated or analysed during this study are included in this published article.

## Abbreviations

AMD: Age-related macular degeneration
ASC: Adipose stem cell
BDNF: Brain derived neurotrophic factor
BMSC: Bone marrow derived mesenchymal stromal cell
BRB: Blood retinal barrier
CAC: Circulating angiogenic cell
CB: Cord blood
DM: Diabetes mellitus
DME: Diabetic macular edema
DR: Diabetic retinopathy
DRIL: Disorganisation of retinal inner layers
ECFC: Endothelial colony forming cell
EPC: Endothelial progenitor cell
ERG: Electroretinogram
ESC: Embryonic stem cell
FAZ: Foveal avascular zone
FFA: Fundus flourescein angiography
FITC: Fluorescein isothiocyanate
GCC: Ganglion cell complex
GFAP: Glial fibrillary acidic protein
HbA1C: Glycated haemoglobin
hESC: Human embryonic stem cell
hiPSC: Human induced pluripotent stem cell
HREC: Human retinal endothelial cell
I/R: Ischemia–reperfusion
iBRB: Inner blood retinal barrier
iPSC: Induced pluripotent stem cell
IRMA: Intraretinal microvascular abnormalities
MAC: Myeloid angiogenic cell
mfERG: Multifocal electroretinogram
MPC: Mesenchymal progenitor cell
MeeSC: Mesenchymal stromal cell
NG2: Neuron-glial antigen 2
NGF: Nerve growth factor
NPDR: Non-proliferative diabetic retinopathy
oBRB: Outer blood retinal barrier
OCT: Optical coherence tomography
OCT-A: Optical coherence tomography angiography
OIR: Oxygen induced retinopathy
ONL: Outer nuclear layer
PDGRF-β: Platelet-derived growth factor receptor beta
PDR: Proliferative diabetic retinopathy
rd1: Retinal degeneration 1 mouse model
rd10: Retinal degeneration 10 mouse model
RGC: Retinal ganglion cell
RPC: Retinal progenitor cell
RPE: Retinal pigment epithelium
SD-OCT: Spectral-domain optical coherence tomography
STZ: Streptozotocin
TEER: Transendothelial electrical resistance
VEGF: Vascular endothelial growth factor
VP: Vascular progenitor
α-SMA: α-Smooth muscle actin
